# Probiotics for Alzheimer’s Disease: A Systematic Review

**DOI:** 10.3390/nu14010020

**Published:** 2021-12-22

**Authors:** Ruth Naomi, Hashim Embong, Fezah Othman, Hasanain Faisal Ghazi, Nithiyah Maruthey, Hasnah Bahari

**Affiliations:** 1 Department of Human Anatomy, Universiti Putra Malaysia, Serdang 43400, Malaysia; ruthmanuel2104@gmail.com; 2 Department of Emergency Medicine, Faculty of Medicine, Universiti Kebangsaan Malaysia, Kuala Lumpur 56000, Malaysia; hashimembong77@ukm.edu.my; 3 Department of Biomedical Sciences, Faculty of Medicine and Health Sciences, Universiti Putra Malaysia, Serdang 43400, Malaysia; fezah@upm.edu.my; 4 College of Nursing, Al-Bayan University, Baghdad 10071, Iraq; dr.hasanainhabasha@gmail.com; 5 Department of Physiotherapy, Faculty of Health and Life Sciences, Inti International University, Persiaran Perdana BBN Putra Nilai, Nilai 71800, Malaysia; nithiyah.maruthey@newinti.edu.my

**Keywords:** probiotics, Alzheimer, mechanism, clinical trials, beneficial, brain-gut axis

## Abstract

Alzheimer’s disease (AD) is the most common form of neurodegenerative disorders affecting mostly the elderly. It is characterized by the presence of Aβ and neurofibrillary tangles (NFT), resulting in cognitive and memory impairment. Research shows that alteration in gut microbial diversity and defects in gut brain axis are linked to AD. Probiotics are known to be one of the best preventative measures against cognitive decline in AD. Numerous in vivo trials and recent clinical trials have proven the effectiveness of selected bacterial strains in slowing down the progression of AD. It is proven that probiotics modulate the inflammatory process, counteract with oxidative stress, and modify gut microbiota. Thus, this review summarizes the current evidence, diversity of bacterial strains, defects of gut brain axis in AD, harmful bacterial for AD, and the mechanism of action of probiotics in preventing AD. A literature search on selected databases such as PubMed, Semantic Scholar, Nature, and Springer link have identified potentially relevant articles to this topic. However, upon consideration of inclusion criteria and the limitation of publication year, only 22 articles have been selected to be further reviewed. The search query includes few sets of keywords as follows. (1) Probiotics OR gut microbiome OR microbes AND (2) Alzheimer OR cognitive OR aging OR dementia AND (3) clinical trial OR in vivo OR animal study. The results evidenced in this study help to clearly illustrate the relationship between probiotic supplementation and AD. Thus, this systematic review will help identify novel therapeutic strategies in the future as probiotics are free from triggering any adverse effects in human body.

## 1. Introduction

Probiotics are living microorganisms that promote health benefits when consumed in adequate quantity. They regulate the level of pH in the body, help preserve the integrity of the intestinal lining, act as antibiotics, and enhance the brain-derived neurotrophic factor [1]. These neurotrophic factors are made up of a type of protein in the brain that facilitates the survival and differentiation of neurons. Hence, it plays a crucial role in neurological development. Learning disabilities and memory impairments are some of the common issues that tend to arise if these factors are missing from the brain [2]. The effects of probiotics on the central nervous system (CNS) are achieved by alteration of gut microbiota, by increasing the diversity of the good bacterial composition, thereby boosting CNS functions. Apart from brain neurotrophic factor, probiotics tend to provide good prognosis in curing memory deficits and psychiatric disorders by directly modifying brain biochemical components such as serotonin, γ-aminobutyric acid (GABA) and dopamine [3].

Probiotics have been proven to restore the homeostasis of gut microbiota and delay the progression of AD, particularly inflammatory reaction and oxidative stress, thereby ameliorating cognitive decline [4]. In this context, prebiotics and synbiotics are quite new and have been studied in a very lesser extent [5]. In regards to this claim, authors compile recent evidence on the role of probiotics in AD. Most commonly used probiotic microorganisms belong to the genera *Lactobacillus* and *Bifidobacterium*. Since they are free from lipopolysaccharides, they do not induce any form of inflammation upon ingestion [6]. *Lactococcus, Streptococcus, Enterococcus, Bacillus clausii* and *Enterococcus faecium SF68* are also used in probiotics. In these, *Escherichia coli* Nissle 1917 strain is a novel probiotic that synthesises via semirough lipopolysaccharide (LPS), which do not have P and S fimbrial adhesions. Absence of these features make it special by making it non-pathogenic, thus an ideal microorganism for probiotics as well [7].

### Alzheimer’s Disease

Alzheimer’s disease (AD) is a progressive neurodegenerative disorder that accounts for 80% of dementia worldwide, particularly in elderly people over 60 years of age. [8]. A systematic analysis for the Global Burden of Disease study shows that about 43.8 million people worldwide suffered from AD in 2016 [9]. According to the world AD prediction in 2016, over 131 million will suffer from AD by the year 2050, making it one of the major global health challenges in the future [10]. AD is characterised by significant memory, cognitive, and motor impairments. This is mainly due to the presence of tau tangles within the neurons or beta amyloid (Aβ) plaque deposition outside the neuron. This may lead to alteration in calcium homeostasis, neuroinflammation, and vascular degeneration, eventually leading to neuronal death. Loss of neuron, synaptic dysfunction, and neuropil threads are closely associated with AD [11].

The pathogenesis of AD is mainly correlated with the amyloid precursor protein (APP), an integral transmembrane in protein processing pathway. Mutations in APP, Presenilin 1(PSEN1) and Presenilin (PSEN 2), are the primary causes of the early onset of AD. Other factors, such as lifestyle, aging, diet, environment, and overexpression of Apolipoprotein (Apo) E4 gene, contribute to the late onset of AD [12]. Accumulation of Aβ is a common pathology in AD. Aβ is a peptide derived from the proteolytic cleavage of APP. APP is transported from trans-Golgi networks through clathrin-mediated endocytosis into the endosomal compartment. During this, a fraction of APP is recycled back to the cell surface in the endosome [13].

APP on the cell surface is regulated by a non-amyloidogenic pathway in which α-secretase acts at the N-terminal end within the Aβ domain. Resultantly, this forms APP-α and membrane-tethered 83 amino acids comprising of carboxy terminal end (CTF). Thus, CTF-83 will be further cleaved to form the intracellular domain of APP and P3 fragment by γ-secretase [14]. APP in the endosomal compartment will enter into the amyloidogenic pathway. The β-secretase interacts on the extracellular domain of APP in this pathway, producing membrane-bound 99 amino acid comprising of CTF-β (C99) and APP-β. γ-secretase will further cleave C99 to form soluble Aβ fragment and APP intracellular domain [15]. Oligomerization of Aβ peptide takes place in the presence of transition metal ions such as Fe^2+^ and Cu^2+^, which produce H_2_O_2_. This will stimulate lipid peroxidation, which eventually forms 4-hydroxynonenal (4HNE) [16].

On the other hand, impairment in the transport of glucose and glutamate increases the influx of Ca^2+^, causing inositol 1,4,5-trisphosphate (IP3) synthesis that induces Ca^2+^ efflux from endoplasmic reticulum storage. Activation of the calcium-dependent calpain triggers cyclin-dependent kinase (CDK5), causing tau hyperphosphorylation, progressing to neurofibrillary tangle (NFT) formation, leading to microtubule disassembly and impairment of the axonal transport. This eventually leads to neuronal and synaptic dysfunction, causing neuronal death [17]. The increased influx of Ca^2+^ ions within the mitochondria and reactive oxygen species (ROS) stimulates the formation of mitochondrial cyclophilin D. Thus, proapoptotic factors such as apoptosis inducing factor (AIF) and cytochrome c will be released. In due course, caspase cascade will be activated leading to neuronal cell death [18].

In addition, Aβ plaques trigger the microglial cells to release the chemokine such as macrophage inflammatory protein-1, IL8 and cytokines such as IL-1, IL-6 and TNF-α. All of these in turn stimulate the release of cytokines, chemokine, and acute-phase protein from astrocytes, activating the microglial cells. The activated astrocytes and microglia generate neuroinflammation in the brain, serving as a pathology in AD [19]. Recent data indicates that a high level of peripheral immune cells present in AD subjects and presence of peripheral immune cells clearly participate in local inflammation. Regardless of the source of peripheral inflammation, whether adiposity or via systemic inflammation, these pro-inflammatory cytokines cells can readily penetrate into blood brain barrier (BBB) and further trigger brain-specific inflammatory responses. Resultantly, BBB becomes more porous and permeable, allowing the entry of peripheral immune cells. In turn, excessive microglial cells will be activated. This will initially evince as impaired hippocampal-dependent learning in AD patients [20].

## 2. Methods

### 2.1. Search Strategy

The literature search is done based on preferred notification items for systematic reviews and meta-analyses (PRISMA) guidelines [21]. Suitable articles relevant to the title of this review are identified. Boolean operator guideline has been utilised to identify the relevant keywords [22]. This includes 3 sets of keywords such as, (1) Probiotics OR gut microbiome OR microbes AND (2) Alzheimer OR cognitive OR aging OR dementia AND (3) clinical trial OR in vivo OR animal study. The search for literature is limited to publications from January 2010 onwards up to October 2021.

### 2.2. Inclusion Criteria

All research articles based on in vivo and clinical trials are chosen to be further reviewed. The chosen articles must be written in English only, and contain an abstract. The selected articles further screened for compatibility. It must contain either all or one of the following measures. These include (1) probiotic strains, (2) targeted microbes, (3) duration of treatment, (4) signalling mechanism, and (5) findings.

### 2.3. Exclusion Criteria

All forms of thesis dissertations, case reports, review articles, and patents are excluded from being further reviewed. Those articles published beyond year 2010 or those that did not meet the inclusion criteria have been excluded from further analysis. Besides, studies that discuss mild cognitive impairment or dementia have been excluded too. For clinical trials, only studies that concentrate on the patients who have been confirmed with AD are selected to be further reviewed. Articles based on synbiotics or prebiotics for AD have been excluded from being further analysed.

### 2.4. Data Extraction and Management

**Figure 1:**
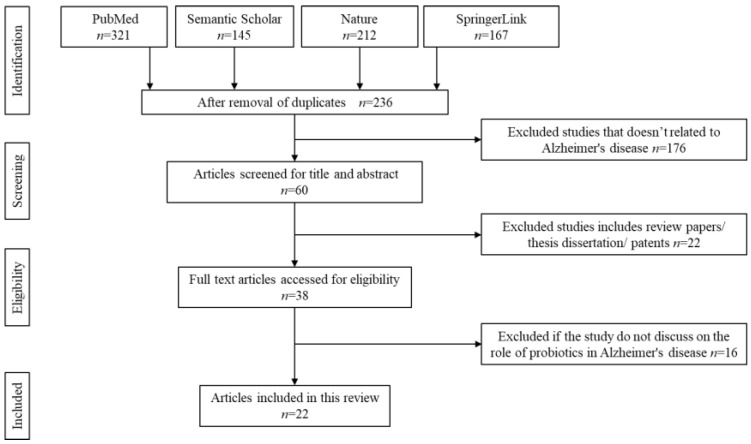
Identification and screening for literature search.

The screening for literature is done based on the Figure 1. As a primary step, the title and abstract of each article is screened independently by four reviewers (R.N., H.F.G., H.E., and F.O.). All the chosen articles must have discussed on one of the following measures stated in the inclusion criteria. Any disagreement is resolved through discussion and consultation with a fifth reviewer (H.B.). Upon agreement, a standardised form is used to note down for data extraction purposes. The data extraction form consists of the following details: (1) author information and year of publication, (2) type of study, (3) duration of treatments, (4) types of subjects, and (5) major findings observed. For in vivo studies, no duplication or dispersion measurements are described. In vivo studies are found to be bias-free, whereas clinical trials are subjected to a bias evaluation.

### 2.5. Strategy for Data Extraction

The findings of the selected articles are provided in the data extraction table below (Table 1 and Table 2) while the study outcome is discussed in the results section. The analysis of the results has been extensively explained in the discussion section.

## 3. Results

### 3.1. Literature Search

Initial literature search identified 845 potentially relevant articles. Due to duplication and irrelevance to the topics, approximately 609 articles are removed. The titles and abstracts of all the remaining articles are thoroughly screened. About 176 articles are removed as the content does not focus on Alzheimer’s disease. Another 22 articles are automatically rejected if they include under patents, secondary literature, case reports, or thesis dissertations. From the remaining 38 articles, 16 articles are rejected upon full text screening if they do not meet the inclusion criteria of this study. A total of 22 articles are finally selected to be further reviewed. The flow chart of the screening, identification, and the reasons for exclusion are summarised in Figure 1.

### 3.2. Characteristics of the Included Studies

We identified 18 animal studies and 4 clinical trial studies that have reported the potential effects of probiotics in Alzheimer’s disease. Table 1 and Table 2 describe the selected studies according to the type of probiotics, their effects, and mechanism of actions.

## 4. Discussion

### 4.1. Role of Gut Microbiota in the Etiology of Alzheimer’s Disease

Up until now, the exact pathogenesis of AD remains unclear. However, there is growing evidence that revealed the involvement of gut microbiota in the neuropathology of AD. Gut microbiota interact with the pathogenesis of AD via several pathways: neuroinflammation, Aβ abnormality, tau phosphorylation, neurotransmitter dysregulation, and oxidative stress. These pathways are dysregulated following a derangement in the microbiota composition and associated with the increase in BBB permeability that promotes neuroinflammation, neuronal cell loss, and ultimately AD [23].

A large body of evidence has accumulated on the important role of neuroinflammation in the pathogenetic process of AD. The brain consists of various cells, including neurons, astrocytes, microglia, oligodendrocytes, and endothelial cells. Inflammation that occurs in the brain is triggered by abnormal accumulation of inflammatogenic molecules through the activation of innate and acquired immunity. Several experimental studies have reported the interaction between the gut microbiota with the expression of central immune cells. Germ-free animals treated with antibiotics demonstrate impairment in microglial maturation and immune response to bacterial stimuli [24]. Alterations in the gut microbiota can activate proinflammatory cytokines and increase intestinal permeability, which lead to the translocation of Aβ oligomers from the intestine to the brain. Sun and co-workers (2020) inject the gastric wall of mice with Aβ1-42 oligomers and observe that the amyloid contributes in causing neuroinflammation and AD [25]. This inflammatory environment can potentiate neuroinflammation and brain dysfunction via systemic inflammation-derived proinflammatory cytokines.

Aβ aggregation in the brain remains the most important pathogenic process of AD. Studies have reported that several bacteria populating in the gut microbiota are able to produce a significant amount of monomeric soluble LPS and Aβ, which might play in the modulation of signalling pathway that will affect the host immune and nervous system [26]. Any breakdown in intestinal barrier function will lead to the activation of immune cells by the interaction between gut bacteria-derived LPS and toll-like receptor 4 (TLR4) signalling pathway [27]. The soluble form of Aβ might polymerize over time producing insoluble fibrous protein aggregates that might be responsible in the pathogenetic process of AD. Several studies also report the correlation changes between gut microbiota composition with Aβ deposition in the brain. Li and co-workers (2020) report changes in gut microbiota of APP_SWE_ mice are correlated with increase expression on amyloid precursor protein and amyloid deposition by stimulating the MAPK signalling pathway [28]. MAPK pathways mediate Aβ induced astrocyte activation, which is the crucial hallmark in the pathogenetic process of AD [29].

Microglia, which is the main myeloid cell in the brain, is maintained by host microbiota under steady state conditions to prepare for the innate immune response in the CNS. Erny and co-workers (2015) observe defects in microglia properties of a germ-free mouse as a result of loss of a complex host microbiota [24]. Evidence suggests that reactive microglia provide a protective barrier around amyloid deposits, preventing the accumulation of new Aβ onto existing plaques [30]. However, some of the microglial activities become static in chronic inflammation and disrupt its ability to clear off the amyloid deposition. Minter and co-workers (2016), show that antibiotic treatment in APP_SWE_ mice alters the composition of gastrointestinal microbiome and correlates with the reduction in the Aβ deposition [31]. Additionally, Aβ secreted by the pathogens induces the release of pro-inflammatory cytokines such as interleukin IL-17A and IL-22, and these cytokines can cross the blood brain barrier (BBB), trigger immune activity, and participate in chronic neurodegenerative disease such as AD [32].

Several studies evaluate the gut microbiota perturbation on tau protein phosphorylation in the pathoetiology of AD. Tau is a microtubule-associated protein that is abnormally phosphorylated to make up the paired helical filaments of neurofibrillary tangles in AD. Vogt and co-workers (2018) report that the gut microbiota-derived metabolite, Trimethylamine N-oxide (TMNO), is higher in the cerebrospinal fluid (CSF) of individuals with mild cognitive impairment and AD [33]. The CSF TMNO can promote and enhance the assembly of hyperphosphorylated tau protein, which is potential contributor in the characteristic of AD pathology [34]. Wang and co-workers (2015) explore possible correlation between *Helicobacter pylori* and Alzheimer-like tau hyperphosphorylation [35]. The study finds that the *H. pylori* increases the tau hyperphosphorylation which is attenuated by the synthase kinase-3β (GSK-3β). The GSK-3β signalling pathway has been recently implicated in the outer membrane vesicle-induced tau hyperphosphorylation, leading to cognitive impairment [36]. The study by Kim and co-workers (2020) finds that frequent transfer and transplantation of fecal microbiota from WT mice into AD-like pathology with amyloid and neurofibrillary tangles (ADLP^APT^) transgenic mouse model ameliorates tau pathology and memory impairment [37].

Neurotransmitters including Acetylcholine (Ach), GABA, dopamine, histamine, noradrenaline, and serotonin (5-HT) can modulate immune system pathways that influence behaviour, memory, and learning in neurodegenerative disorders. In fact, gut bacteria have been found to have the capability to produce neurotransmitters and play an important role in modulating the gut-brain axis. Recent post-mortem brain of AD patients concluded that GABA and glutamate neurotransmitters level are considerably reduced, indicating deficient synaptic function and neuronal transmission in AD [38]. Another study reports the causal effect of elevated GABA, which is a downstream product of Blautia- dependent arginine metabolism with a lower risk of AD [39]. GABA, the major CNS inhibitory neurotransmitter is also produced by the families *Bifidobacterium*, *Lactobacillus*, *and Streptococcus* [40]. 5-HT is a neurotransmitter synthesized from the degradation of amino acid, tryptophan, and plays a key role in regulating appetite, mood, sleep, and sexual function. A recent study provides evidence that high dietary fibre intake upregulates the expression of 5-HT and inhibits neuroinflammation [41]. In the gut, 5-HT is produced by *E.coli*, *Streptococcus* sp., and *Lactobacillus* sp. [40]. Catecholamines, such as noradrenaline and its precursor, are produced by pathogenic *Escherichia coli*, *Proteus vulgaris*, *Serratia marcescens*, and *Bacillus* species [40]. Dopamine can be produced by Staphylococcus in the human intestine by converting the precursor L-3,4,-dihydroxy-phenylalanine (L-DOPA) [42]. Alteration of the norepinephrine and dopamine have been reported in AD patients, whereby both catecholamines concentration are decreased [43].

The brain of AD patients demonstrates an increase in oxidation during the course of the disease. Gut microbiota may influence the level of oxidative state in AD, either by interfering with the level of reactive oxygen species (ROS) or antioxidant system. Gut *Lactobacilli* and *Bifidobacteria* can convert nitrate and nitrite into nitric oxide (NO), which becomes noxious under conditions of oxidative stress. The oxidative reductive reaction of NO will form toxic compounds known as ROS, which are associated with mitochondrial dysfunction and neuronal apoptosis [44]. Oxidative stress can accelerate Aβ deposition and trigger oxidative reaction [45]. In the study by Kanamaru and co-workers (2015), they report the enhancement of oxidative stress and Aβ deposition in double transgenic mouse model of AD [46]. This oxidation is suggested to be the pathological marker in the disease’s progression of AD patients by increasing Aβ, tau hyperphosphorylation, and neuronal death [47]. Although the comprehensive etiopathogenesis of AD remains unclear, understanding the roles and mechanisms of gut microbiota in the AD pathogenesis can help develop promising strategies in the AD treatments.

### 4.2. Deficient of Probiotics in Alzheimer’s Disease

Probiotics alterations greatly influence the progression of AD. In this case, it is proven that in AD there is a drastic reduction in bacteria belonging to the genera *Verrucomicrobia*, *Actinobacteria, Firmicutes,* and *Proteobacteria*. At the same moment, it is common to notice a rise in genera *Tenericutes* and *Bacteroidetes* [48]. This distinct microbial constitution enhances the deposition of Aβ in cerebral [49]. Similarly, a clinical study reveals that composition of AD subjects’ gut microbiota shows reduced microbial diversity and changes in bacterial abundance including decreased levels of *Firmicutes* and *Bifidobacterium*, and increased levels of *Bacteroidetes* [48]. However, the exact mechanism on the influence of probiotics in AD still remains elusive. Researchers have speculated this might be due to the brain-gut-microbiota axis mechanisms [49].

Alteration in gut microbiota may result in the colonisation of intrinsic pathogens. As a consequence, the permeability of the gut will increase, which might disrupt the gut-brain axis mechanism. In regards to this statement, the presence of enterobacteria further modulates the progression of AD by enhancing immune hemocyte recruitment to the brain. This triggers neurodegeneration mediated by TNF-JNK in AD [50]. In fact, certain intestinal opportunistic bacteria, namely, *Escherichia coli, Mycobacterim spp.*, *Salmonella* spp., *Staphylococcus aureus, Klebsiella pneumonia,* and *Streptococcus* spp. have the ability to eliminate microbial exudates and LPS, which are also known as immunogenic components of amyloids [51].

LPS is found within Aβ in the amyloid plaque, suggesting that bacterial constituents can migrate from gut to brain through systemic circulation, thereby further exacerbate Aβ deposition in AD. This triggers a sequence of downstream conditions that led to hindered phagocytosis, causing a rise in the aggregation of Aβ42 [19,22]. Subsequently, certain brain regions such as the cerebellum and the hippocampus will become dysfunctional [52]. Nevertheless, it has been theorised that in AD patients with cognitive disability and brain amyloidosis, an increase of pro-inflammatory gut microbiota such as *Escherichia* spp. or *Shigella* spp. and a decrease of anti-inflammatory taxon like *Enterococcus rectale* have been observed [53]. This highly correlates with the neuroinflammation in peripheral in AD subjects. This clearly indicates that alteration of certain bacterial strain plays a significant effect in the pathogenesis of cognitive deficits and progression of AD severity [54].

### 4.3. Defects of Gut Brain Axis in Alzheimer’s Disease

Gut brain axis refers to dynamic bidirectional interaction between the gut microbiota and the CNS. The interaction between CNS and gut exists through various neuro immune endocrine mediators connecting the peripheral intestinal function with emotional and cognitive brain centres.

The communication between gut and CNS occurs via vagus nerve or hormones produced by endocrine cells such as 5HT [55]. For both mechanisms, the blood-brain barrier acts as a defensive shield by preventing the entry of blood-derived pathogens into CNS. Under normal conditions, soluble Aβ is transported via the receptor for advanced glycation end products from the blood to the brain and cleared through low-density lipoprotein receptors. In AD, a breakdown in the blood-brain barrier is common, which may be attributed to pericyte damage accelerated by the apolipoprotein E gene 4 allele, which is the key genetic risk factor for the late onset AD [56].

Amyloid protein (α-synuclein) is another clinical evidence in AD, particularly in the myenteric neurons of the gut wall. This protein may enter into the neuronal cells from gut lumen through dendritic and epithelial microfold cells in the small intestine Peyer’s patches [57]. In AD, the vagus nerve is severely affected due to the presence of α-synuclein [58]. Along the gut-brain axis, spreading of misfolded proteins (α-synuclein) is common. The aggregation of these misfolded proteins, along with the neuron apoptosis in AD, triggers the release of misfolded proteins into the intracellular space through exocytosis [59]. Subsequently, other neurons will take up these misfolded proteins, which may result in templated conformational changes in susceptible proteins of the cell. The process may spread across neuronal network via synapses. In addition to extracellular Aβ deposition, the protein might accumulate inside neurons. This continues with the formation of neurofibrillary tangles (NFT) deposition of Aβ in extracellular [49].

Nonetheless, the presence of excessive LPS in AD in the perinuclear region further reduces the production of DNA transcription products. This is usually observed within the amyloid plaques and around blood vessels in AD. LPS stimulates TLR in microglial cells that recognise common molecular patterns associated with pathogen damage. Through interactions with cluster of differentiation 14 (CD14) and myeloid differentiation factor 2 (MD-2), LPS induces the inflammatory response of the TLR4 receptor. The activation of TLR4 by CD14 also facilitates inflammatory reaction of Aβ in AD [49].

Aside from this, gut inflammation and dysfunction of gut barrier is common pathology in AD. The presence of intestinal inflammation prompt polymorphonuclear cells to move from the bloodstream to the mucosa of the intestine or even deeper parts of the lumen of the intestine. Elevated levels of Calprotectin in the cerebrospinal fluid of AD are the evidence for this phenomenon. This may pass through the membrane and lead to neuroinflammation. This mechanism is closely associated with dysfunctional gut barrier and leaky gut in AD. In very rare cases, the excess concentration of bacteria in the small intestines does contribute to this leaky gut in AD.

### 4.4. Infectious Hypothesis of Alzheimer’s Disease

**Figure 2:**
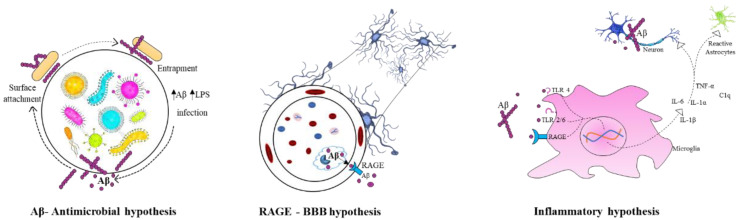
Infectious hypothesis in Alzheimer’s disease.

The infectious theory speculate that the aetiology of AD could be due to the microbes such as virus, bacteria, or prions. The common bacterial species includes *Chlamydia pneumoniae*, *Borrelia burgdorferi*, and *Porphyromonas gingivalis* and human herpes viruses [60]. The mechanism of these microbes invading the brain and leading to AD pathology is still under argument. However, via direct infection, those pathogens could cause neuronal cell apoptosis in CNS. Thus, cognitive impairment and severe inflammation are common observable signs in AD subjects. In such condition, it is well known that inflammation may induce damage to tissues, causing aggregation of Aβ plaques and tangles, thereby promoting further inflammation [61]. This is also known as inflammatory hypothesis. In antimicrobial hypothesis, Aβ attaches to a microbe and entraps it in amyloid fibrils, increasing the formation of amyloid. Whereas, according to receptor for advanced glycation end products (RAGE) at the BBB hypothesis, the influx of circulating Aβ is regulated into the brain via RAGE transporter [62]. Figure 2 summarises the infectious hypothesis of AD.

### 4.5. Mechanism of Action of Probiotics in Alzheimer’s Disease

**Figure 3:**
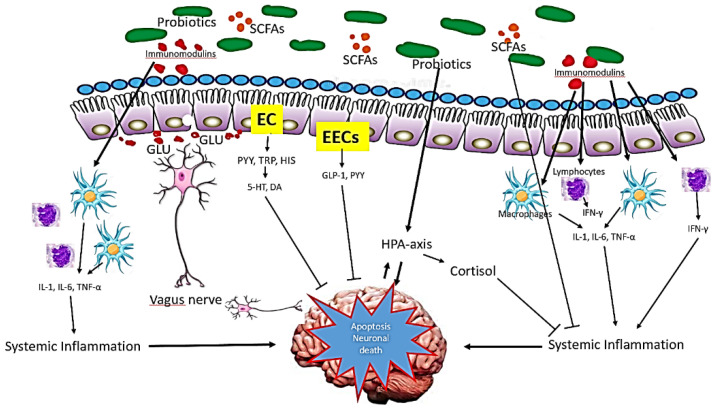
Mechanism of action of probiotics in AD. The probiotics influence brain function via three main function: immune modulation, endocrine pathways and neuronal regulation. Small chain fatty acids (SCFAs), the main metabolites produced by the fermentation of gut microbiota, suppress pro-inflammatory mediators while upregulating the anti-inflammatory mediators. Via endocrine pathways, probiotics activate the hypothalamic-pituitary-adrenal (HPA) axis, stimulate adrenal release of cortisol, which is the most potent anti-inflammatory hormone. Probiotics also stimulate the production of glucagon-like-peptide-1 (GLP-1) and peptide YY (PYY) hormones by intestine enteroendocrine L-cells (EECs). Further, probiotics secrete certain neurotransmitters such as glutamate (GLU) or modulate the secretion of neurotransmitters via enterochromaffin cells (EC) such as serotonin (5-HT). These neurotransmitters and neuroactive metabolites exert neuroprotective effects in concert, preventing neuronal apoptosis.

Probiotics have various mechanisms of action, although the exact manner in which they exert their effects is still not completely elucidated. This occurs from bacteriocin, the production of short chain fatty acids, and competition for nutrients to the stimulation of the role of the gut brain axis and immunomodulation. In regard to this, Figure 3 shows the possible mechanism of action of probiotics in AD.

Short chain fatty acids (SCFAs) are saturated fatty acids produced in the gut depending on the fibre content of the diet. The metabolite include acetate, butyrate, and propionate are produced by fermentation mediated by *Bacteriodes, Clostridium, Lactobacillus, Bifidobacterium,* and *Eubacterium* species [63]. SCFAs might influence brain function via three main pathways: immune modulation, endocrine pathway, and neuronal factors. Via immune modulation, SFCAs enhance barrier integrity and maintain mucus production that can influence intestinal mucosa immunity and barrier function. Systemically, SCFAs mediate immunomodulation through mediation of cytokines’ secretion, which affects proliferation and differentiation of immune cells [64]. This interaction generates anti-inflammatory response and at the same time, suppresses pro-inflammatory cytokines (such as IL-1β, IL-6, TNF-α). In addition, SCFAs can cross the BBB via monocarboxylate transporters and influence the BBB integrity by upregulating the expression of tight junction proteins [65]. In the CNS, SCFAs influence neuroinflammation by affecting microglia cell morphology and function, preventing neuronal cell death.

SCFAs act as an endocrine signalling molecules by modulating the secretion of gut hormones. In murine colonic cultures, acetate and propionate significantly stimulate the secretion of Glucagon-like Peptide -1 (GLP-1) and Peptide YY (PYY) via the G-protein-coupled receptor [66]. GLP-1 is produced and secreted by intestinal enteroendocrine L-cells and neurons within the nucleus of the solitary tract in the brainstem. In the brain, GLP-1 acts as a neuroprotective agent that prevent from neuronal apoptosis and cell death [67]. PYY functions as an appetite suppressing gut hormones. Animal studies demonstrated that neuropeptide Y increases significantly in hippocampus mouse Alzheimer’s disease and exerts neuroprotective effects through activation of PI3K-XBP1-induced Gip78/BiP pathway and inhibition of caspase-3 and caspase-4 activities, suppresses oxidative stress via inhibition of Aβ induced lipid peroxidation and modulates the level of BDNF [68].

Further, SCFAs may modulate the level of neurotransmitters and neurotrophic factors. Studies have shown that gut microbiota can either produce neurotransmitter precursors or catalyse the synthesis and release of several neurotransmitters via dietary metabolism or both [42]. The neurotransmitter precursors stimulate the neurotransmitters production, such as 5-HT and GABA via secretory enterochromaffin (EC) cells. The study by Yano and co-workers (2015) found that butyrate and propionate modulate the biosynthesis of host 5-HT from colonic ECs and serum [69]. The EC cells also produce neuroactive metabolites such as PYY, tryptophan, and histamine. Some neurotransmitter precursors and neuroactive metabolites can cross the BBB and be involved in the synthesis of neurotransmitter in the brain and CNS signalling. Some gut microbiota has a direct influence on the vagal nerve signalling to stimulate the dorsal motor nucleus of the vagus (DMV).

A growing body of research suggests that long-term exposure to stress is a risk factor for Alzheimer’s disease, which may accelerate the illness’s course. The risk of dementia is highly related with stress and worry [70,71,72,73,74,75,76,77]. Stress from the environment or from the outside can lead to psychological distress, which can be compounded by inflammation and oxidative damages. As a result of psychological stress, the hypothalamic-pituitary-adrenal axis (HPA) axis is activated, resulting in the release of glucocorticoids into the bloodstream, which then enters the brain through the blood-brain barrier to activate the glucocorticoid receptor in humans and mineral corticosteroid receptor in mice, respectively [78,79].

Probiotics exert a beneficial effect on the gut-brain-microbiota axis by preventing the hyperactivation of hypothalamic-pituitary-adrenal axis following a gut microbiota dysbiosis and inflammatory processes. Investigation of the different strains of *Lactobaccilus* identified that probiotic *Lactobacillus rhamnosus* decreased the corticosterone levels and anxiety-like behaviour in non-stressed mice [74]. Although HPA dysregulation is believed to be associated with stress, the exact mechanism remains unclear. Probiotic *Bifidobacterium pseudocatenulatum* reduces stress-induced inflammation and improved glucocorticoid sensitivity in murine model of chronic stress induced by maternal separation [75].

### 4.6. Animal Model

Research done on rodents shows memory deficits and cognitive impairment with increased age. Particularly, in AD-induced mice, these functions are severely disrupted. In vivo study as shown in Table 1 reveals that probiotics have effectively modulated gut microbiota, which may enhance cognitive functions related to age in animal models. For instance, an in vivo study done by Nimgampalle and co-workers (2017) on the effect of *Lactobacillus plantarum* MTCC 1325 on D-Galactose-induced AD mice shows decreased level of NFT, amyloid plaques and increased level Ach in the hippocampus and cerebral cortex. At the same time, improvement in memory and spatial learning have been discovered in the mice [76]. This is because the *Lactobacillus plantarum* MTCC1325 strain produces Ach neurotransmitter, which is a potent antioxidant. An increased level of antioxidant will eventually increase the activity of Na^+^ and K^+^ ATPase by reducing acetylcholinesterase (AChE) levels, which then ameliorates the memory by enhancing cholinergic transmission [77]. The rise in Ach level will stabilise the soluble helical content of the secondary Aβ (25–35). The stabilisation will decrease the initiation of Aβ aggregation. In such case, the formation of the toxic oligomer or fibrillar amyloid peptide is hindered [78].

**Table 1:** Effects of probiotics on AD in in vivo studies.

Author (AA)	Probiotics	Animal Model, Sex and Age	Duration	Effects	Mechanism
Nimgampalle et al., [76]	*Lactobacillus plantarum* MTCC 1325	3-month-old male albino rats (Wistar strain)	60 days	Improved spatial memory. Improved gross behavioural activity. Formation of hyperchromatic nuclear chromatin in cytoplasm. Increased level of Ach in hippocampus and cerebral cortex. Decreased level of amyloid plaques and NFT in hippocampus and cerebral cortex.	Production of neurotransmitter such as ACh and AChE.
Asl et al., [79]	*Lactobacillus acidophilus, Bifidobacterium bifidum* and *Bifidobacterium longum*	Adult normal reared male Wistar rats	56 days	Improved spatial learning and memory. Restored synaptic plasticity in brain hippocampus. Prevented accumulation of Aβ peptide in brain hippocampus. Decreased malondialdehyde (MDA) level in brain. Increased total antioxidant capacity in plasma.	Regulation of brain metabolites.
Bonfili et al., [80]	SLAB51	8-week-old male 3xTg-AD mice	16 weeks	Decreased p53 in brain homogenates. Increased level of RARβ levels in brain homogenates. Increased glutathione-S-transferase (GST), glutathione peroxidase (GPx), superoxide dismutase (SOD) and catalase (CAT) in brain homogenates. Decreased poly-ADP ribose polymerase (PARP) in brain homogenates. Decreased 8-Oxoguanine glycosylase (OGG1) brain.	Regulation of brain homogenates.
Bonfili et al., [81]	SLAB51 (*Streptococcus thermophilus, Bifidobacterium longum, Bifidobacterium breve, Bifidobacterium infantis, Lactobacillus acidophilus, lactobacillus plantarum, Lactobacillus paracasei, Lactobacillus delbrueckii subsp. bulgaricus* and *Lactobacillus brevis*)	8-week-old male 3xTg-AD mice	56 weeks	Increased glucose transporter 3 (GLUT3) and glucose transporter 1 (GLUT1) in hippocampal CA1 region. Reduced phosphorylated aggregation of tau levels in brain. Increased HbA1c plasma concentrations. Increased of insulin-like growth factor-I receptor IGF-(IRβ) in brain.	Regulation of brain and glucose metabolism.
Kaur et al., [82]	*Lactobacillus plantarum, Lactobacillus delbrueckii subsp. Bulgaricu, Lactobacillus paracasei, Lactobacillus acidophilus, Bifidobacterium breve, Bifidobacterium longum, Bifidobacterium infantis* and *Streptococcus salivarius subspecies, thermophilus*	6–8-month-old female App^NL−G−F^ and C57BL/6 (wild type)	8 weeks	Increased short chain fatty acids (SCFA) acetate, butyrate and lactate levels in brain hippocampus. Increased serum propionate and isobutyrate. Increased c-fos immunoreactivity in brain. Reduced anxiety-like behaviour.	Regulation of neuronal activity.
Rezaeiasl et al., [83]	*Lactobacillus acidophilus, Bifidobacterium bifidum* and *Bifidobacterium longum*	Male Sprague-Dawley rats	6 weeks	Increased spatial learning and memory. Increased in field excitatory postsynaptic potential (fEPSP) amplitude in hippocampus. Increased of long-term potentiation in CA1 of hippocampus. Decreased paired-pulse facilitation in neurons. Decreased nitric oxide in serum.	Regulation of presynaptic neurotransmitter in brain.
Mehrabadi et al., [84]	*Lactobacillus reuteri, Lactobacillus rhamnosus* and *Bifidobacterium infantis*	Male Wistar rats	10 weeks	Improved spatial learning and memory. Diminished Aβ deposition in hippocampus. Decreased MDA level in brain. Increased SOD in brain homogenates. Decreased IL-1β and α-TNF in hippocampal tissue.	Regulation of brain metabolism.
Kobayashi et al., [85]	*Bifidobacterium breve strain A1*	10-week-old male ddY mice	11 days	Improved spatial learning and memory. Increased plasma acetate level. Suppressed Aβ-induced gene expression in hippocampus. Prevented Aβ-induced cognitive dysfunction.	Down regulation of Aβ-induced gene expression.
Sun et al., [25]	*Clostridium butyricum* WZMC1016	6-month-old APPswe/PS1dE9 transgenic AD model (APP/PS1) mice and wild-type C57BL/6 (WT) mice	4 weeks	Improved spatial learning and memory. Reduced FJC positive cells in the cortex, CA1, and CA2 regions. Decreased Aβ and Aβ42 in brain tissue. Reduced IL-1β and α-TNF in brain. Increased level of butyrate in the faecal. Suppressed the activation of microglia. Reduced expression of COX-2 in brain tissue. Decreased p-p65 level in brain tissue.	Regulation of brain metabolites.
Azm et al., [86]	*Lactobacillus acidophilus* 1688FL431-16LA02, *Lactobacillus* *fermentum* ME3, *Bifidobacterium lactis* 1195SL609-16BS01 and *Bifidobacterium longum* 1152SL593-16BL03	8-week-old male Wistar rats	8 weeks	Increased memory and spatial learning. Increased SOD in hippocampal tissue. Decreased MDA level in hippocampus. Decreased number and size of Aβ plaque in brain.	Regulation of brain metabolites.
Bonfili et al., [87]	*Streptococcus thermophilus, Bifidobacteria longum, Bifidobacteria breve, Bifidobacteria infantis, Lactobacilli acidophilus, Lactobacilli plantarum, Lactobacilli paracasei, Lactobacilli delbrueckii subsp. bulgaricus, Lactobacilli. brevis*	8 week old male 3xTg-AD mice	4 months	Improved cognition and exploratory performance. Increased level of FGF9 in hippocampus. Increased corticol thickness in hippocampus. Reduced plasma concentrations of pro-inflammatory cytokines. Down regulated inflammatory response in plasma. Increased level of ghrelin, leptin, GLP-1 and gastric inhibitory polypeptide (GIP) in the gut. Reduced amount of Aβ1–42 deposits in brain. Decreased level of ChT-L, T-L, and PGPH in the brain homogenates. Decreased level of cathepsin B in brain homogenates. Increased level of cathepsin L in brain homogenates. Increased levels of beclin-1 and LC3-II in brain. Decreased level of p62 in the brain.	Regulation of brain metabolites.
Patel et al., [88]	*Lactobacillus rhamnosus* UBLR-58	Female Swiss albino mice	10 days	Improved memory. Decreased acetylcholinesterase activity in the brain neuron. Decreased MDA level in brain tissue. Increased SOD level in brain. Increased GPx in brain. Increased CAT in brain tissue. Reduced amyloid plaques deposition in brain.	Regulation of brain metabolites.
Cogliati et al., [89]	*Bacillus subtilis* NCIB3610 and *Escherichia coli* OP50	Not relevant	30 days	Increased cognitive function. Decreased Aβ peptide expression. Alleviated behavioural deficits. Increased chemotactic response	
Abraham et al., [90]	*Bifidobacterium longum, Lactobacillus acidophilus* lysates, vitamins A, vitamin D, omega 3 fatty acids in cod liver oil, vitamins B1, B3, B6, B9, B12, and Interval treadmill running	Male APP/PS1 transgenic mice (B6C3-Tg(APPswe, PSEN1dE9)85Dbo/Mmjax; APP/PS1TG)	20 weeks	Increased exploratory activity. Reduced Aβ plaques in hippocampus. Increased microglia in brain. Increased OGG1 levels in brain. Increased Lactobacillus reuteri in gut. Increased cognitive performance.	Regulation of brain metabolism and intestinal microbiome.
Teglas et al., [91]	*Bifidobacterium longum, Lactobacillus acidophilus* lysates, vitamins A, vitamin D, omega 3 fatty acids in cod liver oil, vitamins B1, B3, B6, B9, B12, and Interval treadmill running	3-month-old, male APP/PS1 transgenic mice (B6C3-Tg (APPswe, PSEN1dE9) 85Dbo/Mmjax; APP/PS1^TG^) and six wild types	20 weeks	Increased level of SOD in the hippocampus. Increased level of nuclear factor-erythroid factor 2-related factor 2 (NRF-2) in the liver. Increased 8-oxodG level in hippocampus.	Regulation of brain metabolism.
Yeon et al., [92]	*Lactobacillus helveticus* IDCC3801	Male Sprague Dawley rats and ICR mice	15 days	Increased level of memory and cognition. Decreased level of β-secretase activity in brain. Decreased level of intracellular APPβ. Increased level of intracellular APPα. Decreased level of Aβ40 production in brain.	Regulation of brain metabolism.
Jung et al., [93]	*Lactobacillus Pentosus* and *Lactobacillus plantarum* C29.	Male ICR mice	3 days	Increased spatial learning and memory. Increased level of cAMP response element-binding protein (CREB) in hippocampal. Increased level of BDNF activation in hippocampal.	Regulation of CREB and BDNF.
Wolf et al., [94]	*Lactobacillus casei, Lactobacillus plantarum, Lactobacillus salivarius, Lactobacillus acidophilus, Lactobacillus rhamnosus, Streptococcus thermophilus, Bifidobacterium bifidum, Bifidobacterium infantis, Bifidobacterium longum,* and *Bifidobacterium breve*	7-month-old male and female 3xTg mice	25 weeks	Increased spatial learning and memory. Decreased level of Aβ40 and Aβ42 in hippocampal. Decreased level of amyloid and tau protein in basal forebrain and frontal cortices. Decreased level of microglial marker (ARG1) in brain.	

Another line of study done by Asl and co-workers (2019) shows the similar findings on the effect of *Lactobacillus* and *Bifidobacterium* species on Aβ injected rats. The researcher notices the restoration of synaptic plasticity in brain hippocampus after 56 days of probiotics supplements [79]. *Lactobacillus* and *Bifidobacterium* species restore synaptic plasticity by normalizing the long-term potentiation, which results in increased transmission of signals in the brain. This was followed by significant reduction in microglial marker activation and an increase in BDNF and synapsin expression, showing an improvement in cognition and spatial learning [95]. A study conducted by Bonfili and co-workers (2018) [80] and (2019) [81] reduce Aβ aggregations, preventing the onset and delaying the progression of AD in Tg-AD mice. The result shows that SLAB 51 probiotic ingestion increase expression of GLUT3, GLUT1, and IGF-IRβ by diminishing phosphorylation of activated AMPK and Akt. As a result, tau aggregation starts to reduce [81]. This is because AMPK is known to be one of the positive regulators of autophagy, in which activation of AMPK will enhance the clearance of Aβ through induction of autophagy [96].

Probiotics increase Sirtuin-1 (SIRT1) levels. SIRT1 is a NAD^+^ protein deacetylase with proven neuroprotective action that is capable of reducing ROS levels and promote the survival of the neurons. SIRT1 is inversely associated with acetylated p53, indicating the defensive action of SLAB51 against neuronal apoptosis mediated by p53. SIRT1 is capable to bind with p53 and facilitate survival of cell by suppressing apoptotic response dependent on p53. Through polymerase-1 deacetylation (PARP), SIRT1 inhibits neuronal apoptosis. Probiotic supplements do increase the level of RARβ, which promotes the non-amyloidogenic pathway for APP processing, and the activation is due to deacetylation that favours the transcription of activity of a disintegrin and metalloproteinase 10, which encodes for α-secretase. Resultantly, the generation and deposition of Aβ peptides in the AD brain are able to be prevented [80]. OGG1 binds directly to PARP-1 via the OGG1 N-terminal region, and oxidative stress promotes this interaction [97]. Since probiotic supplements (SLAB51) have the potential to reduce both OGG1 and PARP, it serves as major evidence of reduced oxidised species of DNA in AD mice.

A recent study done by Kaur and co-workers (2020) noted an increased level of SCFA acetate, butyrate, lactate levels, c-fos immunoreactivity (C-fos) in brain hippocampus in VSL#3 supplemented AD mice group. Stoelting testing further confirms the reduction in anxiety-like behaviour in the experimental group [82]. The results indicate that C-fos increases to resemble to the increase of lactate in the hippocampus of the AD mice. C-fos is a transcription factor that determines the functional activity of the neuron in brain. Increased lactate levels modulate the neuronal excitability via ATP-dependent mechanisms involving ATP-sensitive potassium (K_ATP_) channels. As a result, there will be an increase in c-fos level in the brain [82]. C-fos plays a role in modulating stress response, which is the primary reason for the reduced anxiety-like behaviour [98], which is also known to be clinical manifestation in AD induced mice in this study.

Another report by Rezaeiasl and co-workers (2019) shows that probiotic containing the mixture of *L. acidophilus, B. bifidum* and *B. longum* increases excitatory postsynaptic potentials (fEPSPs) in hippocampus and decreases the paired pulse facilitation (PPF) in neurons and NO in serum of Aβ_1-42_ induced mice. PPF is known as a neurotransmitter for presynaptic release and associated with long-term potentiation. The result of the study shows that probiotic increases in the release of presynaptic neurotransmitters, leading to an increase in long-term potentiation on the AD group. This is another reason for relation of probiotic with spatial learning and memory improvement in AD-infused rats [83].

Lab investigations done by Kobayashi and co-workers (2017) [85] and Mehrabadi and co-workers (2020) [84] show almost similar outcome in which the probiotic consisting of *Bifidobacterium breve* strain A1 and *Bifidobacterium infantis* decreases Aβ deposition, IL-1β, and α-TNF and increase SOD level in the brain hippocampus of the Aβ-induced AD mice. *Bifidobacterium* administration inhibits cognitive dysfunction and immune reactive gene expression in the hippocampus by increasing the level of plasma acetate. This proves that *Bifidobacterium* modulates immune response and neuronal inflammation that arise due to Aβ toxicity in the brain tissue. As so, *Bifidobacterium* possesses the ability to suppress toxicity induced by Aβ and normalizes the gene expression profile, particularly BDNF [99], which promotes neuronal survival in AD.

Sun and co-workers (2019) study the effect of *Clostridium butyricum WZMC1016* in APPswe/PS1dE9 mice. The outcome shows a drastic reduction in FJC positive cells in the cortex, CA1, and CA2 regions, COX-2 expression and reduced expression of IL-1*β*, α-TNF and p-p65 in the brain. Simultaneously, suppression of microglia activation is seen in the probiotic infused mice [25]. This indicates that *Clostridium butyricum WZMC1016* is potent to reverse neuron degeneration in the brain and the reduction in FJC+ cells are the evidence for this claim. This claim is further supported by Liu and co-workers in 2015. Through their lab observation, they conclude that *Clostridium butyricum* stimulates anti-apoptotic factors through BDNF-PI3K/Akt pathway in the brain [100]. Nonetheless, the reduced expression of COX-2 will lead arachidonic acid shunting down alternate enzymatic pathways such as cytochrome P450 and epoxygenases, resulting in the synthesis of potentially neuroprotective eicosanoids [101]. Perinuclear aggregates of p65 could be seen in cells containing excess of Aβ or H_2_O_2_. This could be due to the accumulation of covalently modified NF-kB due to increased oxidative stress. Reduction of P-P65 indicates that probiotics supplementation blocked the appearance of Aβ-activated p65 in the AD brain [102].

Research done by Azm and co-workers (2018) on the *Lactobacilli* and *bifidobacteria* strain on Aβ_1–42_ injected rat further proves the positive outcome as per observed in the in vivo trials conducted by other researchers. In these studies, Aβ_1–42_ are injected with 1 × 1010 CFU/g of probiotic containing the mixture of *Lactobacillus acidophilus* 1688FL431-16LA02, *Lactobacillus*, *Fermentum* ME3, *Bifidobacterium lactis* 1195SL609-16BS01 and *Bifidobacterium longum* 1152SL593-16BL03. The outcome is almost similar as other in vivo studies. However, there is significant increase in SOD, and a decreased number and size of Aβ plaque is seen in brain [86]. SOD are the first lines of antioxidant defence against the neurotoxicity of free radicals such as oxidative stress. Oxidative stress may lead to cognitive dysfunction through impaired signalling and neuronal death, leading to neuroinflammation and plaque aggregation. Therefore, antioxidants can be effective in improving cognitive dysfunction, neuroinflammation and clearing of plaques in AD [103]. The improvement of oxidative stress and neuroinflammation by probiotics results in plaque removal and ultimately decreases the number and size of plaques.

Further in vivo trial done by Bonfili and co-workers (2017) on the effect of probiotics in AD mice results in increased levels of cathepsin L, beclin-1, LC3-II, FGF9, and corticol thickness. The level of ghrelin, leptin, GLP-1, and GIP in the gut is also on rise on the 4^th^ month in the experimental group of AD mice. At the same time, significant reduction in ChT-L, T-L, PGPH, and p62 in brain homogenates is recorded [87]. FGF9 increases the proliferation of cells and reduces cell death under oxidative stress starvation. FGF9 substantially upregulates neurotrophic factors derived from the glial cell line and reduces the level of expression of an apoptotic marker, caspase 3 [104]. The effect of probiotics in these studies suggests an increase in the thickness of corticol in the hippocampus. Surzenko and co-workers (2020) find that ingestion of *Lactococcus lactis* increases the proliferation of cortical neural progenitor cells, thereby enhancing the density of cortical neurons that further support this finding [105].

Probiotics have beneficial effects on the regulation of microbiota in the gut. In particular, it has been proven that altering gut microbiome with probiotics promotes increased level of leptin in serum and plasma ghrelin level. Ghrelin is involved in the metabolism of glucose and lipids, which has an effect on mitochondrial respiration; it may also have neuroprotective effects, demonstrating the interaction between metabolism and neurodegenerative mechanisms. Ghrelin and leptin increase neurotrophic factor secretion, thus preventing the toxicity of Aβ oligomers and phosphorylated tau levels in AD mice [106]. Cathepsin B is a cysteine protease associated with amyloid plaques. In AD mice, the probiotic mixture of *Streptococcus*, *Lactobacillus,* and *Bifidobacterium* restores cathepsin L by increasing α-secretase activity, thereby suppressing Aβ levels. In autophagy, beclin-1 plays a crucial role and is involved in the formation of autophagosomes. LC3-II will tightly bind to the autophagosomal membranes. In autophagolysosomes, p62 binds to both LC3-II and ubiquitin and is eventually degraded. Therefore, p62 levels correlate with autophagic activity inversely. The results show that probiotic supplementation increased beclin-1 and LC3-II levels in AD mice and decreased p62 levels, indicating an activation of the autophagic flux [87].

Similarly, Patel and co-workers (2020) witness that *Lactobacillus rhamnosus* UBLR-58 could increase SOD, GPx, and CAT in brain tissue, thereby acting as an antioxidant agent [88]. A study conducted by Cogliati and co-workers (2020) on the efficacy of *Bacillus subtilis* NCIB3610 *and Escherichia soli* OP50 on AD model of *Caenorhabditis elegans* shows favourable results as well. They notice *Bacillus subtilis* could alleviate behavioural deficits and increase chemotactic response [89]. The finding correlates with the outcome seen by Wei Cheng and co-workers (2019), who conclude that *Bacillus subtilis* could stimulate the overproduction of l-tryptophan, increase the level of 5-HT in the hypothalamus, function as an antidepressant and anti-anxiety agent [107].

On the contrary, Abraham and co-workers (2019) [90] and Teglas and co-workers (2020) [91] expose AD-injected mice with probiotic supplementation together with interval treadmill running. Interestingly, they discover that these combination resulted in the increase of *Lactobacillus reuteri* in gut, NRF-2 in the liver, and 8-oxodG level in hippocampus in addition to other outcome as witnessed by other researchers [56,57]. Through gut brain axis communication, *Lactobacillus reuteri* can suppress neuroinflammation in astrocytes in the brain by promoting the production of indole-3-aldehyde and indole-3-propionic acid. These are then transported across the blood-brain barrier [108]. The increase of NRF-2 shows the effect of probiotic in neuroprotection, as NRF-2 provides protection in the neurons via induction of anti-inflammatory, antioxidant, and expression of cytoprotective genes, particularly for oxidative stress in AD [109]. The results of all in vivo studies show that probiotic intervention is successful in improving the inflammatory and oxidative stress in AD-injected mice.

### 4.7. Clinical Trial Effectiveness

The beneficial outcome of probiotics has been widely explored in various fields. However, there is lack of evidence of the modulation of probiotics in patients with AD; impact of probiotics on the onset symptoms and progression of AD still remains unclear. To date, there are only four clinical trials, as shown in Table 2, that have been done on AD patients. For instance, the study done by Akbari and co-workers (2016) proves that probiotic supplement enhances cognitive function and metabolic function in AD subjects. In this study, 30 AD patients received 200 mL/day of milk enriched with probiotics (2 × 10^9^ CFU/g each) for 12 weeks. The obtained result shows an increase in MMSE score, increased insulin tolerance, decreased level of MDA and hs-CRP level in serum, and controlled metabolic status after the 12th week compared to the controlled group. However, there is no effect observed on oxidative stress level [110]. Likewise, Agahi and co-workers (2018) witness similar outcome in their study. In this study, 25 AD patients are provided with probiotics in the dosage of 3 × 10^9^ CFU daily for 12 weeks. In the 12th week, the researchers notice increased TYM score, cognitive function, serum GSH, and decreased level of 8-OHdG in the serum of AD patients. There is no notable effect on total antioxidant capacity [111].

**Table 2:** Effects of probiotics in clinical trials.

Author	Probiotics	Duration	Target	Effects	Mechanism
Akbari et al., [110]	*Lactobacillus acidophilus, Lactobacillus* *cases, Bifidobacterium bifidum, and Lactobacillus fermentum*	12 weeks	-	Improved MMSE score. Reduced serum high-sensitivity C-reactive protein (hs-CRP). Reduced serum triglyceride. Reduced serum MDA. No effect on total antioxidant capacity.	Regulation of metabolic abnormality.
Agahi et al., [111]	*Lactobacillus fermentum, Lactobacillus* *plantarum, Bifidobacterium lactis,* *Lactobacillus acidophilus,* *Bifidobacterium bifidum* and *Bifidobacterium longum*	12 weeks	-	Increased TYM score, cognitive function. No effect on total antioxidant capacity. Increased serum GSH. Decreased serum 8-OHdG.	Regulation of serum metabolites.
Leblhuber et al., [112]	*Lactobacillus casei* W56, *Lactococcus lactis* W19, *Lactobacillus acidophilus* W22, *Bifidobacterium lactis* W52, *Lactobacillus paracasei* W20, *Lactobacillus plantarum* W62, *Bifidobacterium lactis* W51, *Bifidobacterium bifidum* W23 and *Lactobacillus salivarius* W24	28 days	Akkermansia muciniphila Faecalibacterium prausnitzii Clostridium cluster	Reduced concentration of fecal zonulin. Increased Faecalibacterium Prausnitzi in faecal. Increased concentration of kynurenine in serum. Increased concentration of nitrite and neopterin. Increased RNA content in faecal bacteria.	Activation of immune cells leading to stimulation of microbiota gut brain axis.
Tamtaji et al., [113]	*Lactobacillus acidophilus, Bifidobacterium bifidum, Bifidobacterium longum* and selenium	12 weeks	-	Reduced serum hs-CRP. Reduced serum triglyceride. Increased GSH. Increased antioxidant. Improved MMSE score.	Regulation of metabolic abnormality and oxidative stress.

Both results obtained from the study indicate that probiotic is sensitive to the slowing down of AD progression and has positive influence on cognition. This is possible because both *Lactobacillus* and *Bifidobacterium* species have the ability to produce neurotransmitter and neuromodulaters such as acetylcholine, GABA, norepinephrine, serotonin, and dopamine, in which the GABA signalling is linked to cognitive improvements [40]. The increase in GSH, a potent antioxidant, is because the *Lactobacillus* and *Bifidobacterium* stimulate the intracellular GSH production. As a result, lipid, hydroperoxides, hydroxyl radical, and peroxynitrite are neutralised through Se-dependent GPx [114]. Subsequently, the level of 8-OHdG, a biomarker for DNA oxidative damage, MDA, a secondary product of lipid peroxidation in serum of AD patients is reduced. In the brain, the high level of 8-OHdG is closely associated with mitochondrial DNA (mtDNA) loss and mutation, which may induce oxidative stress in AD patients. In this situation, probiotics increase GSH level, thereby GSH protects the mtDNA in the brain cells from hydrogen peroxide (H_2_O_2_) induced damages [115].

Leblhuber and co-workers (2018) notice an increased in kynurenine, nitrite, and neopterin and increase of RNA content of *Faecalibacterium prausnitzii* in the faecal of AD patient upon being supplemented with *Lactobacillus* and *Bifidobacterium*. A reduction in zonulin concentration is observed in the study [112]. Kynurenine reflects immune system activation which corresponds to tryptophan pathway modulation involving indoleamine 2,3-dioxygenase-1. This can be classified as one of the protective mechanisms against inflammation as kynurenin stimulates regulatory T-cells, which dampen the inflammation process, antiproliferative and immunosuppressive activity induced by tryptophan breakdown [60,65,116]. Increased RNA content of *Faecalibacterium prausnitzii* indicates the increase level of anti-inflammatory property. These is because *Faecalibacterium prausnitzii* can produce short chain fatty acids such as butyrate, propionate, and acetate, which plays an important role in gut brain axis [117]. Butyrate is capable of inducing Treg differentiation and control inflammation, decreasing microglial activation and pro-inflammatory cytokines secretion by inhibiting LPS. In contrast, acetate reduces inflammatory signalling through reduced IL-1β, IL-6, TNF-α expression and p38 MAPK, JNK, and NF-κB phosphorylation [118]. Elevation of zonulin concentration, an intestinal permeability biomarker, proves a positive gut brain interaction.

On the contrary, Tamtaji and co-workers (2018) notice a positive correlation between probiotic supplements with antioxidant capacity [113]. This result contradicts with both Akbari and co-workers (2016) [110] and Agahi and co-workers (2018) [111], who state that no effect is observed on antioxidant capacity in the experimental group although they used the same bacterial species for the study. The difference of outcome can be mainly due to the integration of selenium in probiotic supplements. A test done by Ejtahed and co-workers (2012) shows that *Lactobacillus acidophilus* La5 and *Bifidobacterium lactis* Bb12 increase SOD, GPx and total antioxidant in RBC [119]. In regards to these strains, Tamtaji and co-workers (2018) [113] combine selenium in their clinical investigation. The combination of this successfully eliminated radicals such as H_2_O_2_, hydroxyl radicals, and peroxynitrite via cooperation with selenium-dependent GPx [120]. The outcome, witnessed through clinical trials, further confirms the positive effect of probiotic supplements on slowing down AD progression. However, further evaluation is needed to be done in large-scale study to officially prove the effect of probiotic in AD.

## 5. Probiotics as a Therapeutic Target in Alzheimer’s Disease

Based on the animal and clinical trials, the outcome from both investigations shows that probiotic consumption has positive influence on AD. However, a publication biased towards positive results cannot be excluded. Based on the analysis, we can conclude 90% of the study is done based on *Bifidobacterium* and *Lactobacillus*. As so, 100% of the outcome from this study reveals almost similar effects on AD, which is improvement in memory and cognitive dysfunction. Only 10% of the study focuses on *Streptococcus and Clostridium* species. Doses of 10^9^ and 10^10^ CFU have been used in most studies showing an effect on behaviours. Probiotic intake in animals for 2 weeks and in humans for 4 weeks is evidently sufficient to produce significant effects.

The most widely used AD preparations are *Bifidobacteria infantis*, *Bifidobacteria longum*, *Lactobacilli acidophilus*, *Lactobacilli plantarum*, and *Lactobacilli casei*, as single or multi-strain preparations for animal models. All of these probiotics are known to be “good” bacteria, which are likely to prevent the production of harmful bacteria and strengthen the immune system. Probiotics play a vital role in the interaction of gut-brain axis, thereby enhancing both the brain and the gut. Caution is warranted when drawing conclusions from clinical trials that use psychological questionnaires as this may result in subjective biases, rather than clinical or neuropsychological testing for AD patients.

## 6. Faecal Matter Transfer in Alzheimer’s Disease

Faecal matter transplant (FMT) has been suggested to reverse the gut dysbiosis and disorders, improving overall health [121]. FMT, or stool transplant, literally involves directly implanting faecal material from a healthy person to the gut of an inflicted person to restore the diversity of gut microbiota. Recently, FMT has been proposed as a prospective beneficial therapy in treating AD [122]. Hazan (2020) describes improvement in an 82-year-old patient with mild cognitive impairment and recurrent *Clostridium difficile* infection (CDI), who is subjected to 300 mL FMT donated by his wife. The patient’s CDI is resolved and he shows vast improvement in his cognitive alertness even after 6 months of FMT [123]. Park and colleagues (2021) present another case of a 90-year-old woman, with severe CDI and Alzheimer’s dementia, who is also treated with FMT from a healthy asymptomatic donor. The patient’s CDI improved after the second FMT; the patient’s MMSE, which initially is at 15 before FMT, increased to 18 after the first FMT and increased again to 20 after the second FMT indicating improvement in cognition function. The microbiome analysis of the patient indicates change in the bacterial composition after FMT, favouring the abundance of cognitive function associated genera such as *Bacteroidales, Bacteroidia, Tannerellaceae*, and *Actinobacteria* [124].

In animal model, Kim (2020) observes that frequent FMT from wild-type mice to AD-like pathology with amyloid and neurofibrillary tangles (ADLP^APT^) transgenic mouse has decreased the formation of β-amyloid and tau protein in the ADLP^APT^ mice brain [125]. On the other hand, FMT from 5xFAD mice (mice that expressed 5 AD-linked mutation) to C57BL/6 mice is presented with memory dysfunction, reduced neurogenesis, increased TNF-α, IL-1β, and IL-6 indication inflammation of brain and colon [37]. Hence, suggesting that the inflammation of brain is associated with colonic inflammation. Dodiya and colleagues (2019) have demonstrated that FMT from APP/PS1 mice into antibiotic-treated APP/PS1 mice has increased the formation and size of β-amyloid plaque with reduced dendritic points in microglia compared to the control animals [126]. In another study by Fuji el al. (2019), mice transplanted with faecal material from AD patients show earlier loss of cognition function and lower concentration of neurotransmitter such as GABA compared to the control group [127]. Yu and colleagues (2019) learn that memory and spatial learning improvement occur after FMT from Non-CD mice (not CD mice) to pseudo-germ free mice [128].

FMT is a promising therapeutic option against AD. Although numerous successful studies utilizing FMT in reversing cognition function involving animal models are currently available, data on FMT in AD human patients are somewhat limited. Large-scale studies involving human AD subjects are vital in accessing the reliability of FMT in controlling and reversing the detrimental effects of AD.

## 7. Safety Consideration in Probiotics

Probiotics are classified as safe by American Food and Drug Administration (FDA). So far, there has not been any documented evidence on safety issue for *Bifidobacterium, Lactobacillus, Streptococcus,* and *Clostridium* species in AD. However, in certain conditions probiotics should not be administered in AD patients, particularly those who are undergoing immunosuppressive treatments like chemotherapy. Several cases of sepsis, fungemia, and bacteremia have been reported in individuals receiving *S. boulardii.* In rare cases, probiotic bacteria may contain antibiotic resistance genes that they can pass on to other strains of bacteria, including harmful strains that cause infections [7].

## 8. Conclusions and Future Perspectives

In a nutshell, this review provides plenty of evidence based study on the role of probiotics in alleviating the progression of AD. In vivo investigations and clinical trials have proved that probiotics is worth being inculcated in therapeutic field to treat AD. In consideration to this statement, there are no side effects that have been reported for the usage of probiotics for AD. As so, in the future, more clinical trials must be conducted to detect AD-specific changes in the gut microbiome that may provide new insight for probiotics as an ideal therapeutic target. In addition, large-scale studies that associate microbial diversity with cognitive status and progression of disease in AD patients may provide valuable prognostic results. An interdisciplinary approach to investigate the interactions between host and microbiota could potentially lead to a strategic advance in treatment and prevention of AD in future.

## Data Availability

Not applicable.
